# Tumor context determines *ARID1A* effects on gastric cancer immunity

**DOI:** 10.64898/2026.08.18.745460

**Published:** 2026-08-20

**Authors:** Shu Xiao, Ryan T. Heslin, Morgan F. Pettigrew, John D. Karalis, Nafeesah Fatimah, Shao-Po Huang, Verena Cao, Esther Burns, Lynn Y. Kwon, Ibrahim Nassour, Skylar L. Nahi, Hsien Tsung Lai, Changjin Hong, Tae Hyun Hwang, Isaac S. Chan, Suntrea T.G. Hammer, Hao Zhu, Sam C. Wang

## Abstract

The role of *ARID1A* in cancer immune evasion remains uncertain, with prior studies reaching opposing conclusions. In addition, previous work has shown that the role of *ARID1A* in cell-autonomous tumorigenesis is context-dependent. Using isogenic murine gastric cancer models, we found that *in vivo Arid1a* loss in an autochthonous genetically engineered mouse model of gastric cancer conferred T cell-dependent immune evasion, while *in vitro* deletion did not. Mechanistically, tumor *Arid1a* loss reprogrammed the tumor microenvironment into an immune desert through suppression of GM-CSF secretion and interferon-γ responsiveness. These changes were not observed when *Arid1a* was deleted *in vitro* . In human gastric cancer, an immune-cold phenotype was restricted to *ARID1A* mutants in the genomically stable subtype, while *ARID1A* loss in the chromosomal instability subtype was associated with variable immune profiles. These results demonstrate that tumor *ARID1A* loss does not intrinsically confer pro- or anti-tumor immune properties and instead is determined by tissue context.

## Full Text Availability

The license terms selected by the author(s) for this preprint version do not permit archiving in PMC. The full text is available from the preprint server.

